# Early Effects of Scaling Up Dolutegravir-Based ARV Regimens Among Children Living with HIV in Malawi

**DOI:** 10.1007/s10461-024-04312-3

**Published:** 2024-04-13

**Authors:** Lucky Makonokaya, Alice Maida, Louiser Upile Kalitera, Alice Wang, Lester Kapanda, Dumbani Kayira, Madalitso Bottoman, Harrid Nkhoma, Shalom Dunga, Zuze Joaki, Rachel Chamanga, Kondwani Nkanaunena, Susan Hrapcak, Rose Nyirenda, Brown Chiwandira, Martin Maulidi, Godfrey Woelk, Rhoderick Machekano, Thulani Maphosa

**Affiliations:** 1 Elizabeth Glaser Pediatric AIDS Foundation, Lilongwe, Malawi; 2 U. S. Centers for Disease Control and Prevention, Division of Global HIV and TB, Lilongwe, Malawi; 3 U. S. Centers for Disease Control and Prevention, Atlanta, USA; 4 Department of HIV and AIDS, Ministry of Health, Lilongwe, Malawi; 5 Elizabeth Glaser Pediatric AIDS Foundation, Washington, DC USA

**Keywords:** Children living with HIV, Pediatric dolutegravir, Viral suppression, Antiretroviral therapy, Adherence, Malawi

## Abstract

Viral suppression (VS) in children has remained suboptimal compared to that in adults. We evaluated the impact of transitioning children weighing < 20 kg to a pediatric formulation of dolutegravir (pDTG) on VS in Malawi. We analyzed routine retrospective program data from electronic medical record systems pooled across 169 healthcare facilities in Malawi supported by the Elizabeth Glaser Pediatric AIDS Foundation (EGPAF). We included children who weighed < 20 kg and received antiretroviral therapy (ART) between July 2021–June 2022. Using descriptive statistics, we summarized demographic and clinical characteristics, ART regimens, ART adherence, and VS. We used logistic regression to identify factors associated with post-transition VS. A total of 2468 Children Living with HIV (CLHIV) were included, 55.3% of whom were < 60 months old. Most (83.8%) had initiated on non-DTG-based ART; 71.0% of these had a viral load (VL) test result before transitioning to pDTG, and 62.5% had VS. Nearly all (99.9%) CLHIV transitioned to pDTG-based regimens. Six months after the transition, 52.7% had good ART adherence, and 38.6% had routine VL testing results; 81.7% achieved VS. Post-transition VS was associated with good adherence and pre-transition VS: adjusted odds ratios of 2.79 (95% CI 1.65–4.71), p < 0.001 and 5.32 (95% CI 3.23–9.48), p < 0.001, respectively. After transitioning to pDTG, VS was achieved in most children tested within the first 6 months. However, adherence remained suboptimal post-transition and VL testing at 6 months was limited. Interventions to improve VL testing and enhance ART adherence are still needed in CLHIV on pDTG-based regimens.

## Introduction

An estimated 1.7 million children were living with the Human Immunodeficiency Virus (HIV) globally in 2021. Children living with HIV (CLHIV) (< 15 years) have significantly higher morbidity and mortality than adults [1]. In 2021, while CLHIV represented only approximately 5% of people living with HIV (PLHIV), they contributed to 15% of all HIV-related deaths worldwide [2]. Due to their immature immune systems, young CLHIV, in particular, have a more rapid disease progression than adults without effective treatment [3]. There is a need to hasten the transition of CLHIV to more efficacious antiretroviral therapy (ART) regimens to improve their outcomes [4].

Dolutegravir (DTG), a second-generation integrase strand inhibitor, is the third in its class to be approved for the treatment of HIV in children [5–8]. DTG is recommended by the World Health Organization (WHO) as part of the first-line treatment of ART-naïve individuals and treatment-experienced patients [9, 10]. It demonstrates a higher genetic barrier to resistance than other drugs in its class with most of its associated mutations likely to reduce HIV replication and integrase enzymatic activity, especially in ART-naïve patients [11]. It also is a safe and effective drug in adults and children, with patients on a DTG-based regimen achieving viral suppression (VS) as early as 28 days from the onset of treatment [7, 8, 12–14]. Most DTG-based regimens are once-daily doses, making them easier for clients. Many countries quickly adopted DTG-based regimens in adults[3, 10], but pediatric formulations were limited. Children that weighed 30 kg or more were eligible for the once-daily fixed-dose combination tenofovir/lamivudine/DTG, and children between 20 and 29.9 kg were suitable for the adult 50 mg DTG tablets paired with ABC/3TC [10]. However, children < 20 kg remained on either nevirapine-/efavirenz-based regimens, which are suboptimal due to high rates of HIV drug resistance [15], or lopinavir/ritonavir-based regimens, which are poorly palatable, twice-daily regimens [16].

In 2020, the Food and Drug Administration (FDA) and the WHO approved the use of a pediatric formulation of DTG, a 10 mg dispersible tablet, as part of an antiretroviral regimen in infants and children from 4 weeks weighing at least three kilograms [14].

In Malawi, there were an estimated 58,000 CLHIV in 2021, and CLHIV had lower ART coverage than adults living with HIV (74% vs. 92%). CLHIV have lower rates of VS than adults living with HIV in Malawi (54% VS in children versus 85% VS in adult females and 79% VS in adult males) [1]. In 2018, DTG-based regimens were rolled out in Malawi for children, adolescents, and adults living with HIV who weighed ≥ 20 kg [17]. Due to limited VL testing capacity, PLHIV were transitioned to DTG-based regimens regardless of viral load (VL) testing or suppression status [18]. Once the 10 mg film-coated tablet of pDTG became available in 2021 for children < 20 kg, Malawi rapidly transitioned CLHIV to this new optimal formulation.

Despite the availability of pDTG in sub-Saharan Africa since 2021, there needs to be more data from the region on the impact of the introduction and scale-up of DTG-based ART regimens on VS in CLHIV. The Elizabeth Glaser Pediatric AIDS Foundation (EGPAF) in Malawi has supported the scale-up of transitioning children to pDTG-based regimens. This study evaluated the impact of transitioning CLHIV < 20 kg to pDTG-based regimens on VS in EGPAF-supported facilities in Malawi.

### Methods

#### Study Design

EGPAF-Malawi supports 179 ART facilities in nine districts in the central and southern regions of the country, providing HIV care and treatment services to 304,300 PLHIV in 2021. In July 2021, the foundation introduced pDTG to 169 EGPAF-supported facilities, which allowed initiating and transitioning CLHIV weighing between three and 19.9 kg to pDTG-based regimens as first-line ART. We conducted a cross-sectional study among CLHIV receiving ART in the 169 EGPAF-supported facilities in Malawi that provided pDTG to CLHIV.

#### Study Population

We included all children weighing less than 20 kg, and receiving ART in EGPAF-supported facilities between July 2021 and June 2022.

#### Data Collection

We obtained routinely-collected HIV treatment patient-level data for people receiving HIV care in EGPAF-supported ART facilities. We utilized two sources: the Malawi District Health Information Software version 2 (DHIS2) and facility-based ART electronic medical records systems (EMRS). Strategic information and evaluation (SI&E) officers extracted the data using a Microsoft Excel tool.

The outcome variable was VS (defined as < 1000 copies/mL due to varying low detectable limits of VL testing machines), assessed 6 months after initiation or transition to pDTG-based regimens. Independent variables included sociodemographic characteristics (sex, age, guardian type, current district of residence, and the facility location), initial ART regimen, current ART regimen, pre-transition VL results, and adherence to ART which was assessed using pill count. Good adherence was defined as missing ≤ 2 ARV doses/month, whereas poor adherence was defined as missing > 2 ARV doses/month, calculated at the last follow-up visit before the data collection.

#### Statistical Analysis

We performed data analysis using STATA v16 (Stata SE 16.0, StataCorp, College Station, TX, USA). Frequencies and proportions were used to summarize the distribution of demographic characteristics of patients and the characteristics of virally suppressed patients. We employed the Pearson’s Chi square test to compare adherence to ART and the viral load coverage among pediatric patients weighing less than 20 kg 6 months post-transition to pDTG-based regimens by sociodemographic and clinical characteristics. The statistical significance level was 0.05.

We performed a multivariable logistic regression analysis to determine the factors associated with VS among pediatric patients receiving ART in EGPAF-supported facilities who transitioned to pDTG-based regimens. The covariates included in the crude models were the independent variables listed above. Covariates that were statistically significant in the crude models were included in the adjusted model together with sex and age group.

### Ethical Considerations

The Malawi National Health Sciences Research Committee (protocol number 18/09/2130) and Advarra Institutional Review Board (protocol number Pro00032796) in the United States of America (USA) approved the evaluation as part of the protocol titled Evaluation of Outcomes Achieved through Integrated HIV/AIDS and TB Prevention, Care and Treatment Programs in Malawi. We obtained a waiver for informed consent, as only secondary data were utilized for this protocol. This activity was reviewed by the U.S. Centers for Disease Control and Prevention (CDC) and was conducted consistent with applicable federal law and CDC policy.

## Results

A total of 2,468 CLHIV met the inclusion criteria and were included in the analysis. Over half of the CLHIV (56.0%) were female, and the median age was 48 months (interquartile range (IQR) was 33–72 months)(Table 1). Data on initial ART regimen was available for 93.3% of the children: most (89.7%) had been initiated on non-DTG-based regimens; 77.1% had been initiated on a protease inhibitor (PI)-based regimen. Before the transition to pDTG, 59.4% had a VL result; of these, 62.5% had VS. Nearly all patients (99.9%) on non-DTG regimens transitioned to pDTG-based regimens, with the remaining three remaining on PI-based regimens. There was no change in the nucleoside reverse transcriptase inhibitor (NRTI) backbone for children who transitioned to pDTG.

**Table 1:** Demographic characteristics of pediatric patients weighing less than 20 kg receiving ART in 169 EGPAF-supported facilities in Malawi (n = 2468)

Characteristic	Frequency n (%)
*Sex*	
Female	1383 (56.0%)
Male	1085 (44.0%)
Age range (months)	
< 12	45 (1.8%)
12–23	209 (8.5%)
24–59	1110 (45.0%)
60–179	1104 (44.7%)
Facility location	
Urban	780 (31.6%)
Rural	1688 (68.4%)
EGPAF clinical staff available on site	
Yes	1492 (60.5%)
No	976 (39.5%)
Initial ART regimen	
pDTG-based	237 (10.3%)
PI-based^a^	1778 (77.1%)
NNRTI-based^b^	281 (12.2%)
Other	9 (0.4%)
Missing	163
Current ART regimen	
NNRTI-based	0 (0.0%)
PI-based	3 (0.1%)
pDTG-based	2463 (99.9%)
Missing	2
Type of guardian	
Other	246 (10.0%)
Parent	2222 (90.0%)
Pre-transition viral load	
≥ 1000 copies/mL	550 (37.5%)
< 1000 copies/mL	917 (62.5%)
Adherence to ARVs at 6 months follow-up	
Poor adherence	1164 (47.2%)
Good adherence	1304 (52.8%)
Outcome at 6 months follow-up	
Alive in care	2395 (97.6%)
Transferred out	27 (1.0%)
Defaulted	31 (1.3%)
Died	1 (0.1%)
Missing	14

^a^Protease inhibitor-based

^b^Non-nucleoside reverse transcriptase inhibitor-based

Six months after the transition to pDTG, almost all CLHIV (97.6%) were alive and in care. Only approximately half of the patients adhered well to ART (52.8%). ART adherence was better among infants (57.8%) than those aged 24–59 months (48.5%), among patients whose guardians were biological parents (54.0%) than others (42.3%), and among those who had been virally suppressed before the transition to dolutegravir-based regimens (57.0%) than those who had been virally non-suppressed (45.9%). There was no difference in adherence between urban and rural facilities (p = 0.79) or in adherence rates between CLHIV who initiated pDTG- or non-pDTG-based regimens (p = 0.09) (Table 2).

**Table 2:** Adherence to antiretroviral therapy (ART) by sociodemographic and clinical characteristics in children living with HIV (CLHIV) weighing < 20 kg and receiving ART in 169 facilities in Malawi (n = 2468)

Characteristic	Poor adherence n = 1164 (%)	Good adherence n = 1304 (%)	p-value
Sex			
Female	651 (47.1%)	732 (52.9%)	0.90
Male	513 (47.3%)	572 (52.7%)	
Age range (months)			
< 12	19 (42.2%)	26 (57.8%)	< 0.001
12–23	94 (45.0%)	115 (55.0%)	
24–59	572 (51.5%)	538 (48.5%)	
60+	479 (43.4%)	625 (56.6%)	
Facility location			
Urban	371 (47.6%)	409 (52.4%)	0.79
Rural	793 (47.0%)	895 (53.0%)	
EGPAF clinical staff available on site			
Yes	727 (48.7%)	765 (51.3%)	0.05
No	437 (44.8%)	539 (55.2%)	
Initial ART regimen			
pDTG-based	115 (48.5%)	122 (51.5%)	0.09
PI-based	835 (47.0%)	941 (53.0%)	
NNRTI-based	135 (48.0%)	146 (52.0%)	
Type of guardian			
Other	142 (57.7%)	104 (42.3%)	< 0.001
Parent	1022 (46.0%)	1200 (54.0%)	
Pre-transition viral load			
≥ 1000 copies/mL	298 (54.1%)	253 (45.9%)	< 0.001
< 1000 copies/mL	394 (43.0%)	522 (57.0%)	

Only 38.6% of the patients had VL results. More patients receiving care in rural facilities were missing VL results than those receiving care in urban facilities (62.0% vs. 46.6%, p < 0.001). However, there were no differences in VL coverage by sex or age group (Table 3).

**Table 3:** Viral load coverage among pediatric patients weighing less than 20 kg 6 months post-transition to DTG-based regimens in 169 facilities in Malawi (2065)

Characteristic	VL result available n (row %)	Not eligible for VL n (row %)	VL result missing (row %)	p value
Overall	798 (38.6%)	84 (4.1%)	1183 (57.3%)	
Sex				
Female	450 (38.5%)	58 (5.0%)	659 (56.5%)	0.06
Male	348 (38.7%)	26 (3.0%)	524 (58.3%)	
Age range (months)				
< 12	7 (29.2%)	0 (0.0%)	17 (70.8%)	0.56
12–23	48 (35.3%)	7 (5.1%)	81 (59.6%)	
24–59	345 (37.6%)	38 (4.1%)	536 (58.3%)	
60+	398 (40.3%)	39 (4.0%)	549 (55.7%)	
Facility location				
Urban	279 (44.2%)	58 (9.2%)	294 (46.6%)	< 0.001
Rural	519 (36.2%)	26 (1.8%)	889 (62.0%)	
EGPAF clinical staff available on site				
Yes	560 (45.7%)	55 (4.5%)	610 (49.8%)	< 0.001
No	238 (28.3%)	29 (3.5%)	573 (68.2%)	

Approximately 81.7% of the patients with post-transition VL results achieved VS. Factors associated with post-transition VS were good adherence (crude odds ratio [cOR] 1.93, 95% confidence interval [CI] 1.37–2.73, p < 0.001) and pre-transition VS (cOR 5.32 95% CI 3.30–8.57, p < 0.001). In a multivariate analysis, good adherence and VS before the transition remained associated with post-transition VS: adjusted odds ratios (aOR) of 2.79 (95% CI 1.65–4.71), p < 0.001 and 5.32 (95% CI 3.23–9.48), p < 0.001, respectively. There were no significant differences in post-transition VS by sex, age group, geographical location, or guardian type (Table 4).

**Table 4:** Factors associated with viral suppression among CLWH under 20 kg, receiving ART in EGPAF-supported facilities, who transitioned to pDTG-based ART (n = 798)

Characteristic	VL < 1000 copies/mL n = 652 (%)	Crude odds ratios (cOR) (95% CI)	p value	Adjusted odds ratios^a^ (aOR) (95% CI)	p value
Sex					
Female	363 (81.0%)	Ref	–	Ref	–
Male	289 (80.1%)	0.98 (0.70–1.38)	0.90	1.03 (0.62–1.71)	0.90
Age range (months)					
< 12	5 (71.4%)	Ref	–	1	–
12–23	36 (75.0%)	1.89 (0.40–9.02)	0.40	53.29 (0.95–2983.39)	0.053
24–59	268 (77.5%)	2.15 (0.50–9.19)	0.300	14.61 (0.45–472.86)	0.131
60+	337 (84.3%)	3.32 (0.78–14.25)	0.1	17.94 (0.55–586.84)	0.105
Facility location					
Urban	235 (83.3%)	Ref	–	–	–
Rural	411 (79.2%)	0.80 (0.56–1.16)	0.30	–	–
EGPAF clinical staff available on site					
Yes	462 (82.1%)	Ref	–	–	–
No	184 (77.3%)	0.81 (0.56–1.16)	0.30	–	–
Initial ART regimen					
PI-based	548 (81.6%)	Ref	–	1	–
NNRTI-based	95 (76.6%)	0.73 (0.46–1.16)	0.20	–	–
Other	3 (60.0%)	0.34 (0.05–2.03)	0.20	–	–
Type of guardian					
Other	69 (83.1%)	Ref	–	–	–
Parent	577 (80.4%)	0.86 (0.48–1.54)	0.60	–	–
Adherence to ARVs					
Poor adherence	256 (74.4%)	Ref	–	1	–
Good adherence	390 (85.3%)	1.93 (1.37–2.73)	< 0.001	2.79 (1.65–4.71)	< 0.001
Pre-transition viral load					
≥ 1000 copies/mL	171 (71.9%)	Ref	–	1	–
< 1000 copies/mL	336 (92.6%)	5.32 (3.30–8.57)	< 0.001	5.53 (3.23–9.48)	< 0.001

^a^Covariates that were not statistically significant in the crude models were not included in the adjusted model with the exception of sex and age group

Amongst patients with poor adherence (n = 344), 74.4% achieved VS. Amongst the 254 patients who had been virally non-suppressed before the transition to pDTG-based regimens, 71.2% achieved VS after 6 months of pDTG-based ART. Conversely, only 7.4% of the patients who achieved VS on their initial ART regimens before pDTG introduction were virally non-suppressed after 6 months on pDTG-based regimens (Table 4).

## Discussion

Our study demonstrated a successful transition of CLHIV to pDTG-based ART regimens in 169 EGPAF-supported districts in Malawi with improved rates of VS; nearly all CLHIV < 20 kg who had initiated non-DTG-based ART had transitioned to pDTG-based ARV regimens by the end of the study period. This demonstrates the program’s success in transitioning pediatric patients to optimized, pediatric-friendly regimes within the first year of implementation.

Despite improved palatability and once-daily dosing of pDTG-based regimens, ART adherence 6 months post-DTG transition was low, with only about half the patients having good adherence to ARVs, similar to study findings of children on NNRTI- and PI-based regimens from Uganda, Kenya, and Congo [19–21]. ART adherence was better in infants than in older children and patients whose guardians were biological parents than others, similar to a study in Ethiopia that found an association between non-biological caretakers and poor adherence among CLHIV [22]. In our study, good adherence to ART was associated with VS, similar to studies in Vietnam, Uganda, and Thailand, which have demonstrated an increased likelihood of viral non-suppression in those with poor adherence [23–25]. Poor adherence to ART also leads to drug resistance and mortality [26]. These findings highlight the need to explore interventions that help improve adherence to ART among CLHIV, particularly those not living with their parents, including education of non-parental guardians on how to support child adherence. Further information is needed on why adherence rates in CLHIV did not improve with improved palatability and reduction from twice-daily dosing to once-daily dosing, which has been shown to improve adherence in a study conducted in Zimbabwe [26]. Interventions may focus more on other non-medication-related barriers to adherence, such as nutrition status and psychosocial barriers, and patients may benefit from case management and linkage to community-based services.

There was a reduction in VL coverage from pre-transition to post-transition, with only about a third of the patients who transitioned having VL results. This decline in VL coverage post-transition was not unique to CLHIV, as there was a reported overall drop in VL testing in Malawi in 2021, which was partly attributed to viral load bundle supply interruption due to the COVID-19 pandemic [27]. The low post-transition VL coverage limits our understanding of the impact of pDTG on VS. Efforts are needed to increase VL coverage in CLHIV, including stable commodity supplies for sample collection, transport, and VL reagents, adequate sample transportation, and healthcare workers trained in VL sample collection from children.

Although VL coverage post-transition was low, there was an increase in VS rates among those tested, with rates > 80%. These rates are similar to studies conducted in children and adolescents on DTG-based regimens that reported VS [28, 29] but remain below the 2030 95–95–95 UNAIDS targets [25]. However, the proportion of CLHIV with VS was much higher than the Malawi Population-based HIV Impact Assessment (MPHIA) report from a population-based survey conducted in 2015–16 (54% VS) [30]. These findings are encouraging, as improved VS was observed shortly after transitioning to pDTG-based regimens. Nevertheless, as better VS rates are expected on pDTG-based regimens [12], follow-up studies are needed to assess VS in a similar cohort at 12 months on pDTG-based regimens and with higher rates of VL coverage.

Most of the CLHIV, virally non-suppressed on other regimens, achieved VS within the first 6 months of pDTG-based regimens. However, more than a quarter of the patients who had been virally non-suppressed before the transition remained non-suppressed. It is worth noting that all patients who transitioned to pDTG-based ART maintained the NRTI backbone and had one drug replaced. There may already have been viral resistance to the other ARVs leading to functional monotherapy on the dolutegravir-based regimens and possible resistance [12, 31]. However, most children in our study achieved VS with only a one-drug switch and no change to the NRTI backbone. The high rates of poor adherence also make drug resistance less likely to be the main contributor to viral non-suppression in our study. Therefore, further studies that include resistance testing CLHIV on pDTG-based regimens will be essential to understand the contribution of drug resistance in children who do not achieve VS on pDTG-based regimens.

We recognize the following limitations to our study. First, the analysis was based on a retrospective collection of routinely collected data, resulting in incomplete data. Standard program service delivery disruptions, such as VL testing, also affected the study. Also, we could not evaluate other previously known factors associated with viral non-suppression, including malnutrition and co-morbidities [32, 33], as these data are not routinely collected.

## Conclusion

VS was achieved in most CLHIV who received a VL test 6 months after transitioning to pDTG. However, adherence post-transition was suboptimal in this group, and VL testing post-transition was also limited. Interventions that enhance good adherence in children on pDTG-based regimens are required as we progress toward achieving 95–95–95 goals.

## Abbreviations

ART: Antiretroviral therapy
CDC: Centers for Disease Control and Prevention
DTG: Dolutegravir
EGPAF: Elizabeth Glaser Pediatric AIDS Foundation
VS: Viral suppression
VL: Viral Load
CLHIV: Children Living with HIV
NRTI: Nucleoside Reverse Transcriptase Inhibitor
NNRTI: Non-nucleoside Reverse Transcriptase Inhibitor
PI: Protease Inhibitor
MPHIA: Malawi Population-based HIV Impact Assessment
